# Deoxynivalenol-sulfates: identification and quantification of novel conjugated (masked) mycotoxins in wheat

**DOI:** 10.1007/s00216-014-8340-4

**Published:** 2014-12-10

**Authors:** Benedikt Warth, Philipp Fruhmann, Gerlinde Wiesenberger, Bernhard Kluger, Bojan Sarkanj, Marc Lemmens, Christian Hametner, Johannes Fröhlich, Gerhard Adam, Rudolf Krska, Rainer Schuhmacher

**Affiliations:** 1 Center for Analytical Chemistry, Department for Agrobiotechnology (IFA-Tulln), University of Natural Resources and Life Sciences, Vienna (BOKU), Konrad-Lorenz-Str. 20, 3430 Tulln, Austria; 2 Institute of Applied Synthetic Chemistry, Vienna University of Technology, Getreidemarkt 9/163, 1060 Vienna, Austria; 3 Department of Applied Genetics and Cell Biology, University of Natural Resources and Life Sciences, Vienna (BOKU), Konrad-Lorenz-Str. 24, 3430 Tulln, Austria; 4 Present Address: Department of Applied Chemistry and Ecology, Faculty of Food Technology, Josip Juraj Strossmayer University, 31000 Osijek, Croatia; 5 Institute for Biotechnology in Plant Production, Department for Agrobiotechnology (IFA-Tulln), University of Natural Resources and Life Sciences, Vienna (BOKU), Konrad-Lorenz-Str. 20, 3430 Tulln, Austria

**Keywords:** LC-MS/MS, Masked mycotoxin, Phase II metabolism, Wheat (*Triticum aestivum*), Plant-pathogen interaction, Toxicity assessment

## Abstract

We report the identification of deoxynivalenol-3-sulfate and deoxynivalenol-15-sulfate as two novel metabolites of the trichothecene mycotoxin deoxynivalenol in wheat. Wheat ears which were either artificially infected with *Fusarium graminearum* or directly treated with the major *Fusarium* toxin deoxynivalenol (DON) were sampled 96 h after treatment. Reference standards, which have been chemically synthesized and confirmed by NMR, were used to establish a liquid chromatography-electrospray ionization (LC-ESI)-MS/MS-based “dilute and shoot” method for the detection, unambiguous identification, and quantification of both sulfate conjugates in wheat extracts. Using this approach, detection limits of 0.003 mg/kg for deoxynivalenol-3-sulfate and 0.002 mg/kg for deoxynivalenol-15-sulfate were achieved. Matrix-matched calibration was used for the quantification of DON-sulfates in the investigated samples. In DON-treated samples, DON-3-sulfate was detected in the range of 0.29–1.4 mg/kg fresh weight while DON-15-sulfate concentrations were significantly lower (range 0.015–0.061 mg/kg fresh weight). In *Fusarium*-infected wheat samples, DON-3-sulfate was the only detected sulfate conjugate (range 0.022–0.059 mg/kg fresh weight). These results clearly demonstrate the potential of wheat to form sulfate conjugates of DON. In order to test whether sulfation is a detoxification reaction in planta, we determined the ability of the sulfated DON derivatives to inhibit in vitro protein synthesis of wheat ribosomes. The results demonstrate that both DON-sulfates can be regarded as detoxification products. DON-15-sulfate was about 44× less inhibitory than the native toxin, and no toxicity was observed for DON-3-sulfate in the tested range.

**Electronic supplementary material:**

The online version of this article (doi:10.1007/s00216-014-8340-4) contains supplementary material, which is available to authorized users.

**Graphical abstract:**
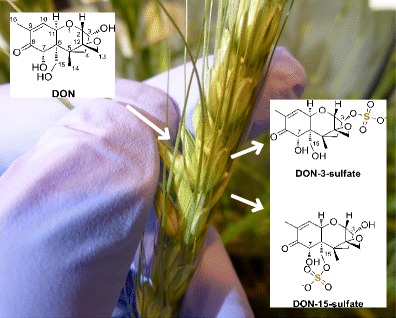
ᅟ

## Introduction

The mycotoxin deoxynivalenol (DON, vomitoxin) frequently contaminates grains and cereal products and thus constitutes a major issue for global food and feed safety. Produced by various *Fusarium* species, this type B trichothecene serves as a major virulence factor of the fungus to invade cereals such as wheat or barley. Its general mode of action is to effectively inhibit protein synthesis [1].

To cope with xenobiotics such as mycotoxins, plants have the potential to modify the chemical structures as part of their defense program. The resulting plant metabolites of mycotoxins are currently neither routinely screened for in food stuff nor regulated by legislation but can be potentially reactivated in the digestive tract and, hence, are considered to be masked mycotoxins [2]. Recently, it was proposed to restrict the use of this term exclusively to the fraction of biologically modified mycotoxins that were conjugated by plants [3]. DON is amenable for metabolism in plants, and several plant conjugates have been described in the literature. The most prominent plant metabolite of DON is DON-3-glucoside (D3G) [2]. Using yeast, *Arabidopsis*, and a wheat germ in vitro translation system, it was shown that D3G formation is a detoxification reaction [4]. Furthermore, the formation of D3G significantly contributes to *Fusarium* resistance in wheat [5]. However, recent human and animal studies indicated that D3G can be effectively hydrolyzed in the intestinal tract resulting in an increase of its aglycon and, hence, posing a potential risk to exposed individuals [6–8]. Therefore, the inclusion of masked mycotoxins in future risk assessment scenarios is warranted. Besides D3G, also DON-diglucoside and oligoglycosylated DON conjugates with up to four bound hexose units were detected in cereal-based products [9]. Recently, an untargeted screening strategy using stable isotopic labelling and liquid chromatography-high-resolution mass spectrometry (LC-HRMS) revealed further DON-biotransformation products including two DON-glutathione (DON-GSH) conjugates and their processing products DON-S-cysteine and DON-S-cysteinyl-glycine in wheat [10]. The increasing interest in conjugated forms of DON and other mycotoxins is also highlighted through the recent establishment of a *Working Group on Masked Mycotoxins in Food and Feed* by the European Food Safety Authority (EFSA) and a request from the European Commission for a “scientific opinion on the risks for animal and public health related to the presence of deoxynivalenol, metabolites of deoxynivalenol, and masked deoxynivalenol in food and feed” [11].

The formation of a sulfate conjugate of the *Fusarium*-produced mycotoxin zearalenone in the model plant *Arabidopsis thaliana* has been demonstrated [12]. *A. thaliana* treated with DON [13] or T-2 toxin [14] showed a more than 20-fold upregulation of a predicted sulfotransferase gene. Also in wheat, a putative sulfotransferase gene was listed under “transcripts exhibiting a negative difference in response for the resistant allele of Qfhs.ifa-5A (adjusted *P* ≤ 0.05, greater than or equal to twofold change, FC) after inoculation with *Fusarium graminearum* spores at 72 h after inoculation” [15]. We therefore set out to test the hypothesis that DON may be converted into a sulfate conjugate. As a first step, the corresponding reference substances were chemically synthesized [16]. In this paper, we present experimental evidence for the existence of two plant-derived DON-sulfate conjugates, which have not been described as plant metabolites before. An LC-MS/MS method for the separation of the two isomers was developed and utilized for quantification purpose.

## Material and methods

### Chemicals and reagents

**Fig. 1:**
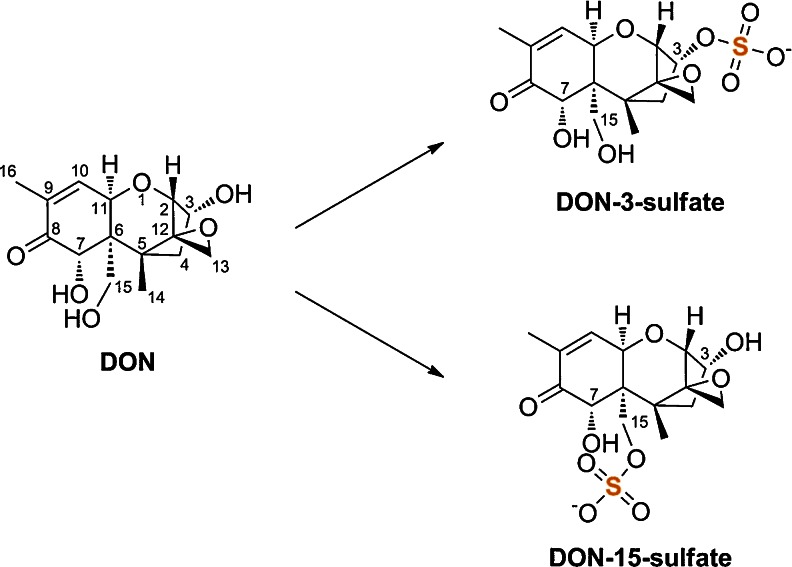
Structure of deoxynivalenol (DON) and its plant metabolites DON-3-sulfate and DON-15-sulfate

Methanol (LC gradient grade) and formic acid (p.a.) were purchased from Merck (Darmstadt, Germany), acetonitrile (LC gradient grade) from VWR (Leuven, Belgium), and ammonium acetate (MS grade) from Sigma-Aldrich (Schnelldorf, Germany). Water was purified using an Elga Purelab ultra analytic system (Veolia Water, Buckinghamshire, UK). Deoxynivalenol-3-sulfate (D3S) and deoxynivalenol-15-sulfate (D15S) (Fig. 1) were synthesized using a sulfuryl imidazolium salt as described by Fruhmann et al. [16]. D3G and DON for analytical purpose were purchased from Romer Labs Diagnostic GmbH (Tulln, Austria), whereas DON for the treatment of plants was produced and purified according to Altpeter and Posselt [17]. Solid substances were dissolved in pure methanol (D3S, D15S) or acetonitrile (DON, D3G) and stored at −20 °C. A combined multi-standard working solution for preparation of calibrants and spiking experiments was prepared in acetonitrile containing 5.0 mg/L of each analyte.

### Samples

The samples used in this study originated from the spring wheat cultivar “Remus,” which is sensitive towards the *Fusarium* head blight disease and the toxic effects of DON. The experiment presented in this publication was part of a larger metabolomics study to investigate the effect of *F. graminearum* and DON on wheat [18]. Results obtained by LC-HRMS, which led to the discovery of GSH-related DON conjugates, have been published elsewhere [10]. Flowering wheat ears were treated either with DON (5 g/L in water), a *F. graminearum* spore suspension (strain IFA65, 10,000 macroconidia/mL), or with water as a control treatment and were harvested 96 h after treatment. Plants were grown, treated, and harvested under standardized conditions as described in detail elsewhere [18]. Five individual biological replicates were carried out for all three treatments. Samples were stored at −80 °C until analysis.

### Sample preparation

Frozen wheat samples were milled individually to fine powder at 30 Hz using a ball mill (MM301 Retsch, Germany) with liquid nitrogen-precooled stainless steel vessels. The homogenized wheat ears (100 ± 2 mg fresh weight) were weighed into Eppendorf tubes and extracted with 1 mL of precooled methanol/water (75 + 25 (*v*/*v*)) including 0.1 % formic acid by vortexing for 10 s and further treatment in an ultrasonic bath at room temperature for 15 min (resulting in a dilution of 1:10 (*w*/*v*)). Samples were centrifuged at 8500×*g* (10 min, 4 °C), and 200 μL of the extract was transferred into a glass vial without any further dilution. For quantification of DON and D3G, which occurred at much higher concentrations, the wheat extracts were diluted to a ratio of 1:100 (*v*/*v*) using methanol/water (50 + 50 (*v*/*v*)) acidified with 0.1 % formic acid resulting in a total dilution of 1:1000 (*w*/*v*). A volume of 5 μL of both dilutions was injected into the LC-MS/MS system in two individual runs.

### LC-MS/MS instrumentation and parameters

Chromatographic separation, detection, and quantification were performed utilizing a QTRAP 6500 system (AB Sciex, Foster City, CA, USA) equipped with an IonDrive™ Turbo V electrospray ionization (ESI) source and interfaced with an Agilent 1290 series UHPLC system (Waldbronn, Germany). Analytes were separated on an Atlantis T3 column (3.0 × 150 mm; Waters, Wexford, Ireland) with 3 μm particle size and a C18 pre-column (Gemini^®^ 4 × 3 mm i.d.; Phenomenex, Torrance, CA, USA). Gradient elution at 30 °C was performed within 14 min. Eluent A (water) and eluent B (ACN) both contained 20 mM ammonium acetate, and the flow rate was set to 600 μL/min. After an initial time period of 0.5 min at 95 % eluent A, the percentage of eluent B was linearly raised to 15 % until 6 min. Then, eluent B was raised to 100 % until 9 min followed by a holding time of 2.0 min and subsequent 3.0 min of column re-equilibration at 95 % eluent A.

ESI-MS/MS was performed in selected reaction monitoring (SRM) mode for all analytes investigated in this study. Two individual transitions were monitored for each analyte. All measurements were done with the following settings: low mass range, source temperature (550 °C), curtain gas (30 psi; 69 kPa of max. 99.5 % nitrogen), ion source gas 1 (sheath gas, 60 psi), ion source gas 2 (drying gas, 60 psi), and collision gas (nitrogen, high). The ion spray voltage was set to −4000 V. Analyte-dependent MS/MS parameters were optimized via direct infusion of the DON-sulfate reference standards. MS/MS spectra (enhanced product ion scans) were recorded at a collision energy of −50 V and a scan rate of 1000 Da/s. For control and data evaluation of HPLC and mass spectrometer, the Analyst software (version 1.6.2; AB Sciex) was applied.

Quantification of the two DON-sulfate isomers was done by matrix-matched calibration curves (1/*x* weighted). To this end, the multi-standard working solution was spiked into blank wheat extracts at six concentration levels. To obtain a suitable blank material for spiking, the water-treated wheat extracts were pooled prior to spiking. External calibration was used for the quantification of DON and D3G.

### Toxicity assessment

In vitro toxicity of DON and sulfate derivatives was examined using a commercial in vitro transcription/translation system (TnT^®^ T7 Coupled Wheat Germ Extract System; Promega, Madison, WI, USA). Standard transcription/translation reactions were performed in a total volume of 15 μL according to the manufacturer’s instructions in the presence of the respective compounds in 0.4 % methanol (final concentration). Ribosomes were first preincubated at 30 °C with inhibitors, buffer, amino acids, and DNA. After 7 min, T7-RNA polymerase was added to start the coupled in vitro transcription/translation reactions, which were stopped after 30 min by adding 1 μL of a 1 mM cycloheximide solution. Efficiency of translation was determined by measuring the activity of the firefly luciferase reporter using the Promega Steady-Glo^®^ Luciferase Assay System and the EnSpire^®^ 2300 Multimode Plate Reader from PerkinElmer. Three independent assays using individual dilutions were performed for each substance.

## Results and discussion

### LC-MS/MS method development and performance evaluation

**Fig. 2:**
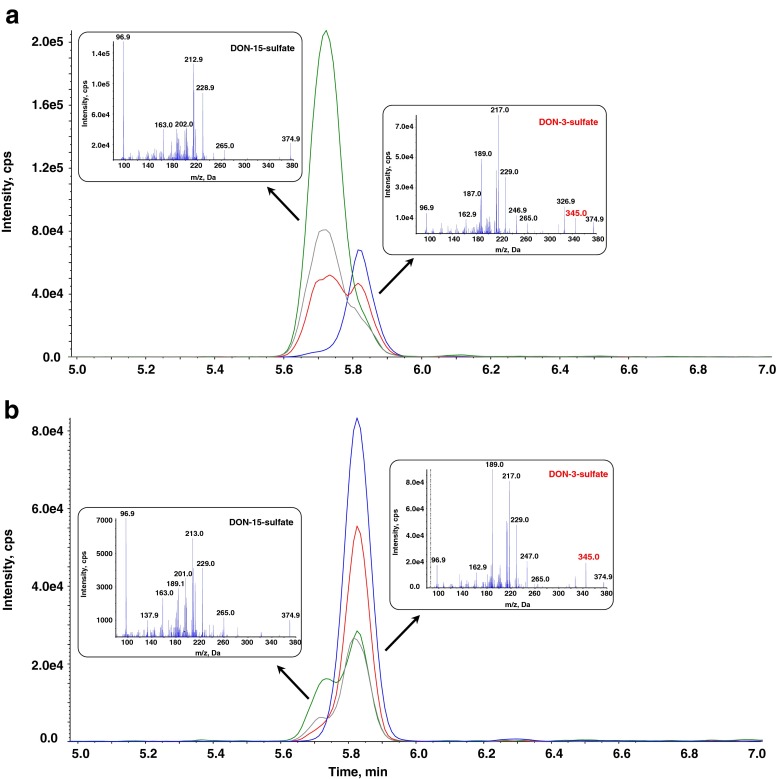
SRM chromatograms and MS/MS spectra of the two DON-sulfate isomers in an authentic reference standard spiked into blank matrix (**a**) and in a wheat sample 96 h after DON treatment (**b**). The standards contained 0.1 mg/L of both sulfates. The concentration of conjugates in the wheat sample was quantified to be 1.4 and 0.061 mg/kg fresh weight for DON-3-sulfate and DON-15-sulfate, respectively. The MS/MS (EPI) spectra of the precursor ion at *m*/*z* 375.0 [M–H]^−^ were recorded at a collision energy of −50 V. The following transitions are displayed: *m*/*z* 375.0→345.0 (*blue*), *m*/*z* 375.0→246.9 (*red*), *m*/*z* 375.0→97.0 (*green*), *m*/*z* 375.0→163.1 (*grey*). The fragmentation product of *m*/*z* 375.0→345.0 (depicted in *red* in the MS/MS) is specific for the DON-3-sulfate isomer

MS/MS parameters of DON-sulfates were optimized in both the positive and the negative ESI mode (Table 1). As expected, both conjugates yielded higher signals in the negative mode. To differentiate between the two isomers, the fragment ion at *m*/*z* 345 (−30 amu) can be used. This corresponds to [M–CH_2_O–H]^−^ with a loss of CH_2_O from the –CH_2_OH group attached to the carbon at the C-6 position of the D3S (see Fig. 1). It cannot be seen in the MS/MS spectrum of D15S (Fig. 2) since the cleavage of the –CH_2_OH group is not possible due to the attached sulfate. Instead, an MS/MS fragment corresponding to [M–SO_4_–CH_2_O–H]^−^ at *m*/*z* 265 is formed by the cleavage of the C6–C15 bond in case of D15S. The same principle was used already in the past to distinguish between the DON-3-glucoside and DON-15-glucoside isomers [19] and the respective DON-glucuronide isomers [20].

**Table 1:** Optimized ESI-MS and ESI-MS/MS parameters

Analyte	*Q*1 (*m*/*z*)	DP (V)	*Q*3^a^ (*m*/*z*)	Relative intensity^b^ (%)	CE^a^ (eV)	CXP^a^ (V)	Dwell time^a^ (ms)
DON	355.1 [M + Ac]^−^	−20	265.0/247.0	32	−20/−22	−17/−17	20/20
DON-3-glucoside	517.0 [M + Ac]^−^	−50	457.1/59.0	5	−18/−50	−10/−10	20/20
DON-3-sulfate	375.0 [M–H]^−^	−125	345.0/246.9	68	−36/−82	−21/−11	50/20
DON-15-sulfate	375.0 [M–H]^−^	−110	97.0/163.1	38	−38/−50	−9/−9	50/20

*DP* declustering potential, *CE* collision energy, *CXP* cell exit potential

^a^Values are given in the order quantifier ion/qualifier ion

^b^Signal intensity of the qualifier transition in relation to the quantifier (Qualifier / Quantifier × 100)

The composition of the eluents was optimized in order to maximize the retention, recovery, and signal intensity of DON-sulfates. Acetonitrile was superior to methanol as eluent B and yielded highest absolute signal intensities when ammonium acetate (20 mM) was added. A similar behavior has been described for other polar DON conjugates as well [21]. Acidification of the eluents resulted in lower signal intensities and was therefore not used in this study.

Matrix-matched calibration was used for quantification since DON-sulfates, especially the D15S isomer, exhibited severe ion enhancement in spiked wheat extracts (data not shown). DON and D3G were quantified by external calibration since the wheat samples were diluted to a ratio of 1:1000 (*w*/*v*) in total, rendering matrix-matched calibration unnecessary due to the high dilution factor and the associated absence of matrix effects. The limits of detection (LODs) and limits of quantification (LOQs) were calculated from the chromatograms of the spiked wheat samples based on a signal-to-noise ratio of 3:1 and 10:1, respectively. The resulting LOD and LOQ values were determined to be 0.003 and 0.01 mg/kg fresh weight and 0.002 and 0.005 mg/kg fresh weight for D3S and D15S, respectively. The analytes eluted after 5.72 min (D3S), 5.82 min (D15S), 6.7 min (D3G), and 7.0 min (DON). All results reported herein were corrected for the dilution of the fresh wheat with extraction solvent (1:10 (*w*/*v*)).

### Identification of DON-sulfates as novel plant metabolites

Both DON-sulfates were detected in DON-treated wheat samples and identified based on comparison with authentic reference standards. These standards were synthesized as potentially interesting DON metabolites in plants, humans, and/or animals by Fruhmann et al. [16] and characterized thoroughly by NMR. In Fig. 2, the SRM chromatograms as well as the MS/MS spectra of D3S and D15S in a reference standard (Fig. 2a) and a DON-treated wheat sample (Fig. 2b) are shown.

To the best of our knowledge, DON-sulfates have not been reported as plant metabolites before. In the literature, only two reports of a DON-sulfate conjugate as an animal phase-II-detoxification product were published. Prelusky et al. [22] detected estimated 2 % of intravenously administered DON as DON-sulfate in sheep urine. However, enzymatic hydrolysis was used to quantify the amount of conjugates which makes the identification and quantification less reliable. Very recently, Wan et al. [23] reported deoxynivalenol-3-sulfate as a major metabolite in chicken tissues following oral administration of labelled DON. The authors putatively identified the metabolite as D3S based on an MS/MS spectrum (including the transition *m*/*z* 345 specific for the D3S isomer), which is indeed very similar to the one obtained using our authentic reference standard (Fig. 2a). However, no verification using (NMR-confirmed) reference standards was performed in the mentioned study [23]. Hence, it cannot be excluded that the MS/MS spectrum was obtained from a mixture of DON-sulfate isomers. During method development, we found out that the Thermo Hypersil column, which was used in the mentioned study [23], is not well suited to separate polar conjugates in general and is hardly able to separate the two DON-sulfate isomers (data not shown). It was proposed that the high sulfation capability of chicken might be one of the reasons for reduced susceptibility towards the toxic effects of DON [23]. Likewise, the sulfation of DON in plants might be a detoxification reaction.

### Sulfation is a detoxification mechanism in wheat

**Fig. 3:**
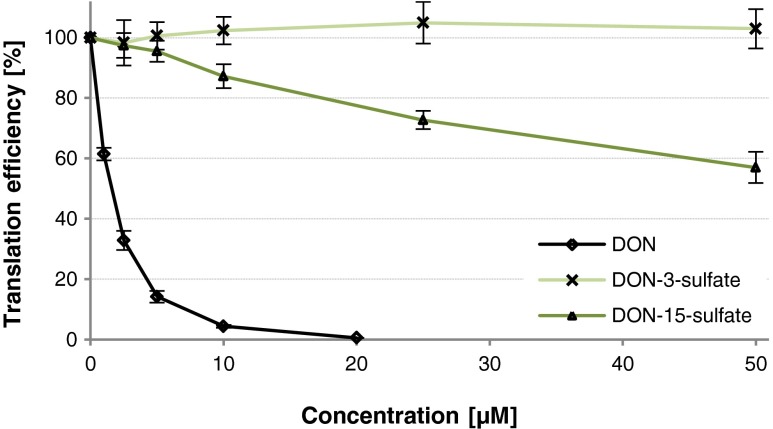
Comparative toxicity of DON and its two sulfate conjugates on the ribosome as determined by an in vitro translation assay with wheat germ extract. *Error bars* indicate the standard deviation of three individual determinations

Since toxicity data were unavailable, we performed in vitro translation assays with wheat germ extract in the presence of DON and two DON-sulfates (Fig. 3). While 1.5 μM DON reduces in vitro translation by wheat ribosomes to 50 % and translation is completely inhibited in the presence of 20 μM DON, DON-3-sulfate does not inhibit in vitro translation at concentrations up to 50 μM. In contrast, DON-15-sulfate is a moderate inhibitor of plant ribosomes with an estimated IC50 of 66 μM (by extrapolation). Thus, it can be concluded that DON-sulfates can be regarded as detoxification products of DON.

It seems plausible that sulfation may be a general strategy of plants to detoxify DON and confers resistance towards the *Fusarium* head blight (FHB) disease (in analogy to DON-3-glucoside as proposed by Lemmens et al. [5]). Future studies should therefore investigate if an effect on the DON/DON-sulfate ratio is evident in near isogenic wheat lines carrying different combinations of FHB resistance-related quantitative trait loci.

### Quantification of sulfate conjugates in wheat

The developed method was applied to quantify the content of DON-sulfates in wheat extracts obtained from wheat ears that were either inoculated with *F. graminearum* or treated with DON directly. Both plant conjugates were detected in all five DON-treated samples. D3S was quantified in the range of 0.29–1.4 mg/kg fresh weight, while D15S concentrations were about a factor of 20 lower (range 0.015–0.061 mg/kg fresh weight). A typical chromatogram of a DON-treated wheat sample is illustrated in Fig. 2b.

Also in *Fusarium*-infected wheat samples, a scenario which is more realistic, small amounts of D3S were detected (range 0.022–0.059 mg/kg fresh weight). In the control group (water treatment), neither DON nor any of its plant metabolites were detected. The complete results for all the tested samples are reported in the Electronic supplementary material (ESM) Table S1. It should be noted that, albeit the surprisingly high concentrations in the mg/kg (or milligram per kilogram) range in DON-treated wheat, the relative concentration of the DON-sulfates to that of the parent toxin DON (0.7 %) and to the major plant metabolite D3G (0.4 %) is generally low. Likewise, in *Fusarium*-infected samples the relative concentration of D3S compared to DON and D3G was 0.1 % and 0.9 %, respectively. However, this might be strongly influenced by the wheat cultivar and the growing conditions and needs to be investigated in more detail.

## Conclusions and outlook

This is the first report on the presence of DON-3-sulfate and DON-15-sulfate in wheat, two novel plant conjugates, and potential masked mycotoxins. An LC-MS/MS-based method was developed for the separation of the two isomers and used to quantify their concentrations in wheat extracts. Furthermore, it was shown that the sulfation of DON is a detoxification reaction in plants. In the future, we aim to investigate the natural occurrence of these novel masked mycotoxins and evaluate if sulfation is a general strategy of plants to detoxify DON which might confer resistance towards the *Fusarium* head blight disease. Furthermore, the toxicity of DON-sulfates for animal cells and its metabolic fate in the digestive tract of humans and animals should be assessed in order to evaluate their potential impact on future DON risk assessment.

## Electronic supplementary material

Below is the link to the electronic supplementary material.

ESM 1 (PDF 19.2 kb)
