# Supplementary material for: Deoxynivalenol-sulfates: identification and quantification of novel conjugated (masked) mycotoxins in wheat
Source: Anal Bioanal Chem. 2014 Dec 10;407(4):1033–9. doi: 10.1007/s00216-014-8340-4 (PMC4305104; doi:10.1007/s00216-014-8340-4)
Supplement: Supplementary file 1 — (PDF 19.2 kb) [file 216_2014_8340_MOESM1_ESM.pdf]

Analytical and Bioanalytical Chemistry

Electronic Supplementary Material

Deoxynivalenol-sulfates: identification and quantification of novel conjugated (masked) mycotoxins in wheat

Benedikt Warth, Philipp Fruhmann, Gerlinde Wiesenberger, Bernhard Kluger, Bojan Sarkanj, Marc Lemmens, Christian Hametner, Johannes Fröhlich, Gerhard Adam, Rudolf Krska, Rainer Schuhmacher

Table S1 Concentrations of deoxynivalenol, its glucoside and its sulfates in the analyzed wheat samples (n=10) 96 hours after inoculation with *Fusarium graminearum* (Fg) or direct treatment with the toxin (DON). The reported concentrations refer to the wheat's fresh weight (f.w.)

	DON-3-sulfate [mg/kg f.w.]	DON-15-sulfate [mg/kg f.w.]	DON [mg/kg f.w.]	DON-3-glucoside [mg/kg f.w.]
Fg_1	0.055	< LOD	35	9.5
Fg_2	0.059	< LOD	23	6.3
Fg_3	0.038	< LOD	25	5.3
Fg_4	0.032	< LOD	24	4.8
Fg_5	0.022	< LOD	10	2.5
**Average Fg treatment**	**0.041**	**< LOD**	**24**	**5.7**
**Max. Fg treatment**	**0.059**	**< LOD**	**35**	**9.5**
DON_1	1.1	0.039	1.2×10^2	1.9×10^2
DON_2	0.90	0.039	1.0×10^2	2.1×10^2
DON_3	1.3	0.050	1.4×10^2	2.2×10^2
DON_4	1.4	0.061	1.7×10^2	2.3×10^2
DON_5	0.29	0.015	0.54×10^2	8.6×10^2
**Average DON treatment**	**1.0**	**0.041**	**1.2×10^2**	**3.4×10^2**
**Max. DON treatment**	**1.4**	**0.061**	**1.7×10^2**	**8.6×10^2**
